# Thermally reduced pillared GO with precisely defined slit pore size†

**DOI:** 10.1039/d0ra00067a

**Published:** 2020-02-13

**Authors:** Andreas Nordenström, Artem Iakunkov, Jinhua Sun, Alexandr V. Talyzin

**Affiliations:** Department of Physics, Umeå University Umeå Sweden Alexandr.Talyzin@umu.se; Department of Industrial and Materials Science, Chalmers University of Technology Gothenburg Sweden

## Abstract

Graphene oxide (GO) pillared with tetrakis(4-aminophenyl)methane (TKAM) molecules shows a narrow distribution of pore size, relatively high specific surface area, but it is hydrophilic and electrically not conductive. Analysis of XRD, N_2_ sorption, XPS, TGA and FTIR data proved that the pillared structure and relatively high surface area (∼350 m^2^ g^−1^) are preserved even after thermal reduction of GO pillared with TKAM molecules. Unlike many other organic pillaring molecules, TKAM is stable at temperatures above the point of GO thermal reduction, as demonstrated by TGA. Therefore, gentle annealing results in the formation of reduced graphene oxide (rGO) pillared with TKAM molecules. The TKAM pillared reduced graphene oxide (PrGO/TKAM) is less hydrophilic as found using dynamic vapor sorption (DVS) and more electrically conductive compared to pillared GO, but preserves an increased interlayer-distance of about 12 Å (compared to ∼7.5 Å in pristine GO). Thus we provide one of the first examples of porous rGO pillared with organic molecules and well-defined size of hydrophobic slit pores. Analysis of pore size distribution using nitrogen sorption isotherms demonstrates a single peak for pore size of ∼7 Å, which makes PrGO/TKAM rather promising for membrane and molecular sieve applications.

## Abstract 2

The porous structure of tetrakis(4-aminophenyl)methane (TKAM)-pillared graphene oxide preserves after thermal reduction providing rare example of true pillared reduced GO material with precise slit pore size and sizable surface area.

## Graphical abstract

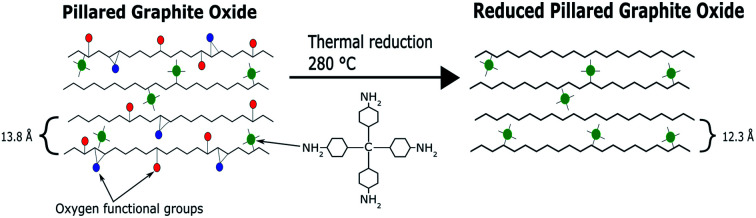

## 1. Introduction

Graphite oxide (GO) is a layered, non-stoichiometric material. It is prepared by strong oxidation of graphite and is composed of carbon, oxygen and hydrogen. The layered structure of graphite is preserved after oxidation but the interlayer distance increases from ∼3.4 Å to ∼7 Å due to the addition of oxygen functional groups on the basal planes.^1^ Several structural models have been proposed for GO over the years and its exact structure has been the subject of considerable debate.^2–5^ In fact, GO is not one material but many materials with strong difference in oxidation procedures, degree of oxidation, relative amount of various functional groups and defect state. As a result, many properties of GO are rather different depending on the details of preparation, *e.g.* Brodie *vs.* Hummers oxidation procedures.^6^ Some properties of GO are also affected by impurities specific to the method of synthesis, most importantly sulphur in Hummers GO.^7^

It is generally accepted that all kinds of GO show non-stoichiometric composition and complete structural disorder in location of oxygen functional groups. Epoxy and hydroxyl functional groups are usually considered as the most abundant on basal planes, while carboxyls and carbonyls are found on the edges of GO sheets.^5,8–10^ However, the relatively high amount of double bonded oxygen functionalities typically found in Hummers GO, for example, using XPS also requires the presence of holes or point defects in the carbon lattice.^11–13^

Thanks to the reactive oxygen functionalities GO provides broad possibilities for chemical modification.^10,14^ For example covalent functionalization of GO with amines has been reported in several studies.^15–20^ One of the most important properties of GO is swelling in polar solvents, which can be considered as an example of non-covalent functionalization.^1,21^ The expansion of the GO lattice due to swelling provides “permeation channels”, which is important in membrane applications of multilayered graphene oxide materials.^22–24^ Numerous attempts to control the interlayer spacing using intercalation of certain cations into GO membrane lattice have been reported.^25^ The swelling properties of GO are drastically different for GO produced using Brodie and Hummers oxidations.^12^ It also depends on pressure-temperature conditions and is strongly affected by the nature of solvents, providing inter-layer distances in the range of ∼9–50 Å.^26–29^

Swelling is also an important step in the preparation of “pillared” GO structures (PGO).^30^ The preparation of PGO typically includes three steps:

(1) The molecules dissolved in polar solvents penetrate between GO layers into a swelled structure

(2) “Pillars” are formed by the attachment of molecules to the carbon lattice by covalent or non-covalent functionalization

(3) Solvent is removed by evaporation while molecular pillars keep graphene oxide layers apart preserving average distance typical for swelled structure.

Examples of molecules reported to be suitable for preparation of PGO include diboronic acid,^31–33^ 1,4-diethynylbenzene^34^ and diaminoalkanes.^35^ Recently, we reported PGO pillared with rigid tetrapod-shaped 3D amine molecules (TKAM).^30^ Remarkably, the TKAM-pillared material could only be prepared using GO synthesized by Hummers' method but not using Brodie GO (BGO).^30^ BGO is a distinctly different material.^36^ The swelling of BGO in methanol and ethanol at ambient temperature provides smaller inter-layer distance below 10 Å (ref. 12) which is insufficient for penetration of relatively large TKAM molecules. Swelling of HGO in methanol and ethanol is stronger (13–14 Å at ambient conditions^12,27^) thus providing expansion of lattice sufficient for the insertion of TKAM molecules as required for pillaring reactions.^30^

The PGO structures typically exhibit relatively high surface area and precisely defined inter-layer distance between GO flakes forming subnanometer sized slit pores.^30,31,37^

It is important to distinguish between the true pillared GO structures and intercalated GO. The term “pillaring” has been used rather often for materials with expanded GO lattice but negligible (or not reported) Specific Surface Area (SSA). In our opinion, the expansion of GO lattice due to intercalation should not be considered as a pillaring until evidence for the existence of interconnected pore network is provided. Many reports on pillared GO or pillared graphene structures do not provide any information about surface area of materials.^17^ In other cases the “pillared” materials demonstrate rather low surface area,^38,39^ the lattice expansion does not match with the size of pillaring molecules^40^ or pore size determined using nitrogen sorption is not in agreement with proposed pillared structures.^41^

Here we define pillared GO structure as a material with the lattice expanded by pillaring molecules providing interconnected pore network accessible for the penetration of gases or liquid solvents.^30^ The interconnected pore network is most easily detectable using analysis of nitrogen sorption isotherms which need to demonstrate sizable value of SSA. Considering our definition of pillared structures, there are rather few examples of true PGO materials with typical BET SSA values of ∼200–1000 m^2^ g^−1^.^32^

Pillared GO have been proposed for applications in energy storage,^42^ gas storage,^31,43^ as membranes,^17^ and as electrode material for supercapacitors.^44,45^ However, PGO's typically inherit the insulating nature of GO, which is a problem for certain applications, *e.g.* for application as electrode materials. So far, this problem has been addressed by using reduced graphene oxide, often in combination with conducting pillaring molecule.^40,45–48^

The typical approach to create pillared structures is to use rGO dispersions and to add pillaring molecules in process of assembling 3D structure. However, this approach typically leads to rather irregular packing of rGO layers, most often amorphous structure, as observed by XRD, and relatively small SSA values. Moreover, it is known that rGO powder typically exhibits SSA of a few hundreds of m^2^ g^−1^ even without pillaring, providing rather broad pore size distribution.^49^ Therefore, it is often impossible to assign the surface area only to pillaring and ruling out the surface area of rGO powder precursor.

Here we explore an alternative approach for the preparation of pillared rGO. To the best of our knowledge, there has been no reports on pillared GO prepared using reduction of the pillared GO structure. Using chemical reduction will almost always destroy the PGO structure while using thermal reduction is limited by relatively low temperatures of degradation for many organic “pillars”. The remarkable thermal stability of TKAM pillaring molecules provides a rare opportunity to reduce GO using simple thermal treatment while keeping the pillared structure intact.

Here we report the preparation of a novel pillared material obtained by thermal reduction of GO pillared with TKAM molecules. It is demonstrated that the TKAM pillared rGO (PrGO/TKAM) remains porous, exhibiting interlayer distance of ∼12 Å by XRD, pore size distribution with single peak ∼7 Å and sizable surface area thus providing the first example of true pillared rGO structure directly obtained by the reduction of pillared PGO.

## 2. Experimental

### 2.1 Materials and preparation

Commercial graphite by Alfa Aesar (natural powder, 325 mesh, 99.8%) was used as starting material for the preparation of graphite oxide samples following the Hummers' method.^50^ Using standard characterization, *i.e.* XRD, FTIR, TGA and XPS, it was determined that the GO samples were of high quality. A C/O of 2.3 was determined by XPS. Hummers GO always contain some traces of sulphur contamination, but careful washing allowed to reduce the sulphur content to 0.36%.

Commercial tetrakis(4-aminophenyl)methane (TKAM) from Sigma Aldrich was used as a pillaring agent.

The TKAM pillared GO (PGO/TKAM) was prepared according to previously reported solvothermal procedure.^30^ Precursor GO powder (typically 400 mg) and TKAM (1 : 1 by weight) were mixed in a stainless steel reactor with methanol (typically around 80 mL). The reactor was closed under nitrogen and annealed at 110 °C for 24 h. The reaction mixture was centrifuged (4400 rpm for 15 min) in order to separate the powder from the remaining solution. The unreacted TKAM molecules were removed by repeated washing with methanol. Typically ∼500 mL of methanol was used for the washing procedure. The sample was finally dried under vacuum at room temperature for at least 12 h. TKAM pillared reduced GO (PrGO/TKAM) was obtained by thermal annealing of PGO/TKAM powder at 280 °C under argon flow for 45 min with heating rate 1 °C min^−1^ (starting from room temperature). Two batches of the material were prepared with reproducible results.

### 2.2 Characterization methods

The as-synthesized PGO/TKAM and PrGO/TKAM samples were characterized by X-ray photoelectron spectroscopy (XPS), thermogravimetric analysis (TGA), BET, X-ray diffraction (XRD), Fourier transform infrared (FTIR) and dynamic vapor sorption (DVS). The nitrogen sorption isotherms were measured using a Autosorb iQ XR and Nova 1200e surface area & pore size analyzers (Quantachrome) at liquid nitrogen temperature. The relative pressure interval *P*/*P*_0_ for the BET plot was selected using a procedure optimized for microporous materials.^51^ A slit-pore QSDFT equilibrium model was applied to evaluate pore volume and pore size distribution.

Panalytical X'pert X-ray diffractometer with Cu-Kα radiation (*λ* = 1.5418 Å) was used to record the diffraction patterns. XPS spectra were recorded with a Kratos Axis Ultra electron spectrometer equipped with a delay line detector. A monochromatic Al Kα source operated at 150 W, a hybrid lens system with a magnetic lens, providing an analysis area of 0.3 × 0.7 mm, and a charge neutralizer were used for the measurements. The binding energy scale was adjusted with respect to the C1s line of aliphatic carbon, set at 285.0 eV. All spectra were processed with the Kratos software. TGA was done by using a Mettler Toledo TGA/DSC1 STARe System. Experiments were performed from room temperature up to 800 °C at a heating rate of 5 °C min^−1^ under nitrogen flow (50 mL min^−1^).

Dynamic vapor sorption (DVS) data were recorded using a DVS Advantage® system with SMS UB-1 balance 0.1 μg sensitivity and dry air as carrier gas. All samples were held at 0% humidity during 2 h and heated after for 30 min. Each point of DVS isotherm were recorded until change in the mass was less than 0.005% per min or stopped after at least 900 min of recording.

All data were gathered at 25 °C temperature. The mass signal was collected in range of 0–10% relative humidity with step 1%, 10–20% with 2.5%, 20–50% with 5% and 50–95% with 10% for adsorption and 0–95% with step 20% for desorption. Average time per one isotherm was 3 days.

The conductivity measurements were performed in two steps: first, dry powder was pressed into polypropylene plastic sheet at 10 MPa to create free standing electrodes. These electrodes were typically 10 mm long and 1 mm wide and the layer of active material was around 10–30 μm thick. After the free standing electrodes had been prepared, the conductivity was measured using a Keithley 2450 by 4 point probe measurements.

## 3. Results and discussion

**Fig. 1:**
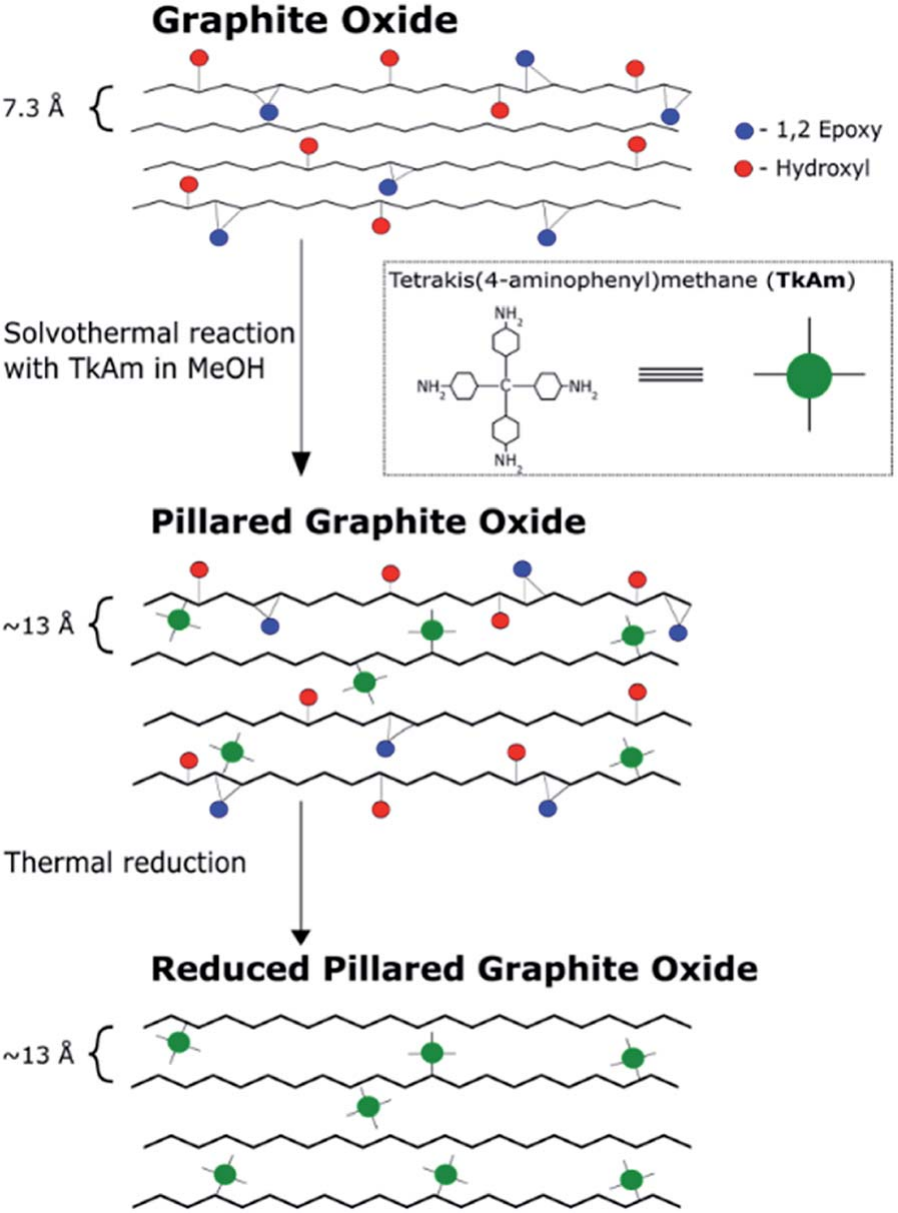
Reaction pathway to create reduced pillared graphite oxide from graphite oxide. Note that the interlayer distance of pillared material (∼13 Å) is based on our previous work.

The main idea of this study was to produce pillared rGO using thermal reduction of PGO. The reduction of GO in the pillared structure can be expected to maintain high surface area measurable by gas sorption methods, improved conductivity, precise slit pore shape, uniform pore size and more hydrophobic properties of the material. GO pillared with TKAM molecules (PGO/TKAM) was used here as a precursor for thermal reduction as shown in Fig. 1.

The precursor PGO/TKAM was prepared using Hummers GO following previously reported solvothermal procedure^30^ with temperature and relative amount of reagents optimized for highest surface area (TKAM : GO ratio of 1 : 1 by weight). The resulting material showed *d*(001) = 13.8 Å significantly increased compared to pristine GO (7.4 Å) (Fig. 3) and sizable BET surface area of 350–530 m^2^ g^−1^ (∼10 m^2^ g^−1^ for precursor GO powder).

Note that maximal SSA values are obtained only after careful vacuum degassing of the PGO/TKAM for at least 24 hours. Moreover, SSA values depends on the temperature of degassing, which must be kept sufficiently low to prevent material from thermal degradation, but high enough to remove most of the adsorbed gases and water. Two sets of measurements demonstrated that even slight change of degassing parameters, temperature from 110 °C (12 h under vacuum) to 120 °C (24 h under vacuum) results in an increase of BET SSA from 350 m^2^ g^−1^ (cumulative SSA 370 m^2^ g^−1^) to 530 m^2^ g^−1^ (cumulative SSA 590 m^2^ g^−1^). However, the increase of SSA is correlated with some loss of oxygen, as detected by XPS, and change in pore size distribution (see Fig. S1†). The combination of expanded GO lattice and relatively high surface area provide evidence for preparation of true pillared GO structure with interconnected pore network, in agreement with our earlier study.^30^

Our earlier studies demonstrated that the main step in thermal decomposition of HGO occurs around 190–200 °C while TKAM is stable at least up to 400 °C.^30^ Therefore, it could be anticipated that annealing of PGO/TKAM in the temperature range ∼200–400 °C might result in thermal reduction of GO sheets while keeping the TKAM pillars preserved. Note that rapid thermal reduction of pristine GO is an explosive process due to the formation of gaseous products but the explosion can be avoided by using slow heating rate.^9^ The pillaring molecules are expected to prevent restacking of rGO flakes into graphitic structure, whereas the interconnected pore network provides a path for escape of evolved gases.

**Fig. 2:**
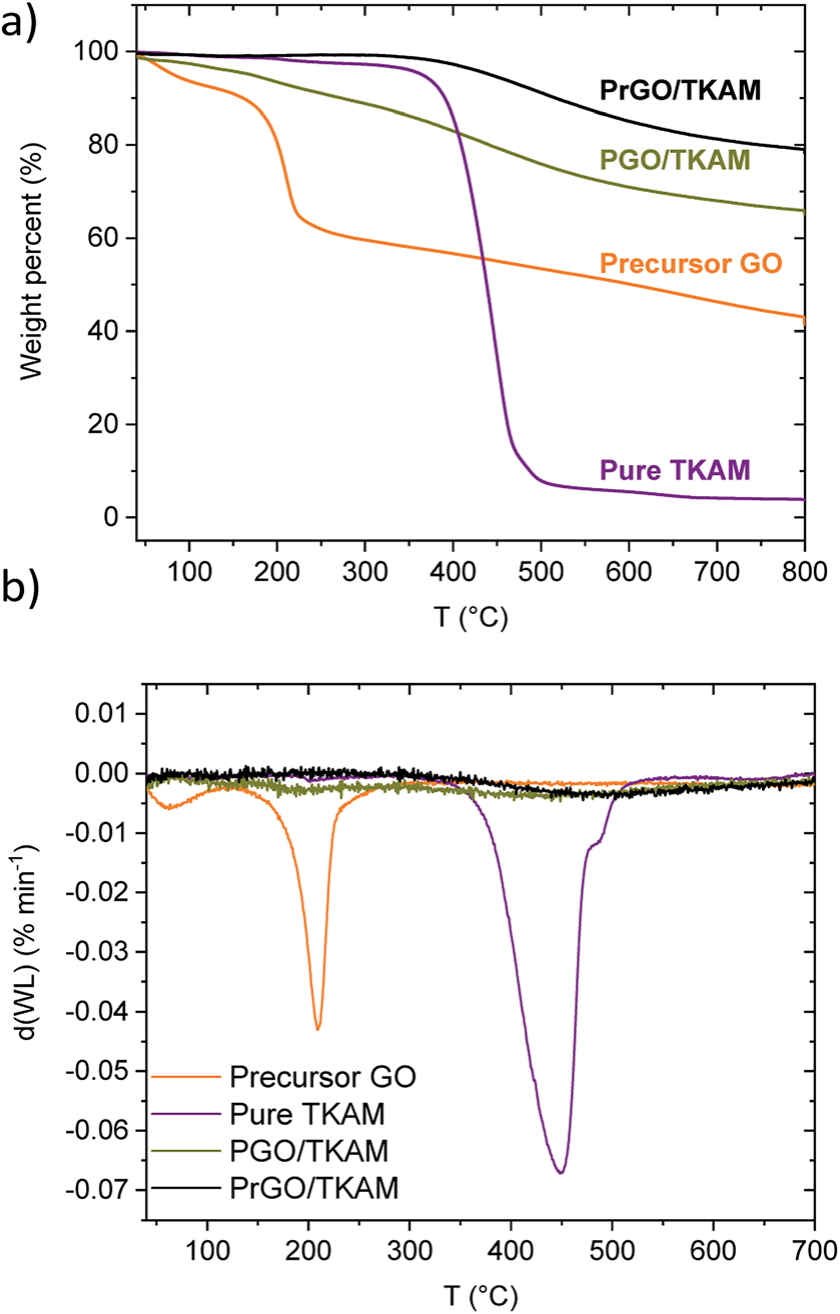
(a) TGA traces of GO, pure TKAM, PGO/TKAM and PrGO/TKAM (prepared by the annealing of PGO/TKAM at 280 °C) and (b) derivate plots for TGA traces.

Fig. 2 shows TGA traces of precursor GO, pure TKAM, PGO/TKAM and PrGO/TKAM (material obtained by thermal reduction of PGO/TKAM). Precursor GO shows weight loss ∼5% due to evaporation of water below 150 °C and major weight loss step ∼30% around 150–250 °C due to deflagration and decomposition (removal of oxygen groups and formation of CO and CO_2_).

The pure TKAM is stable up to ∼400 °C and shows major weight loss at higher temperature due to thermal decomposition of molecules or sublimation. The PGO/TKAM shows weight loss over a broader temperature interval with two steps: first due to deflagration of GO (∼10%) and second due to decomposition of TKAM (∼20%).

The thermal reduction of PGO/TKAM was performed under argon flow using slow heating (1 °C min^−1^) up to 280 °C and annealing at that temperature for 45 minutes. The temperature of 280 °C is higher than the deflagration temperature of GO, but lower than the decomposition temperature of TKAM. Identical heat treatment procedure applied to precursor GO in a reference experiment resulted in formation of trivial rGO. This rGO showed BET SSA of 185 m^2^ g^−1^, and XRD typical for graphitic carbon with *d*(001) = 4.2 Å. The bulk synthesis of PrGO/TKAM resulted in weight loss about 10%, which is consistent with the TGA data (Fig. 2).

The TGA trace of PrGO/TKAM does not show weight loss at temperatures below ∼400 °C typical for graphite oxides confirming that thermal reduction was successful and complete for the given conditions. At the same time the trace of PrGO/TKAM shows weight loss in the temperature region of TKAM precursor degradation. Therefore, the TGA data confirms thermal reduction of PGO/TKAM and provide evidence for the presence of pillaring TKAM molecules in the PrGO/TKAM.

**Fig. 3:**
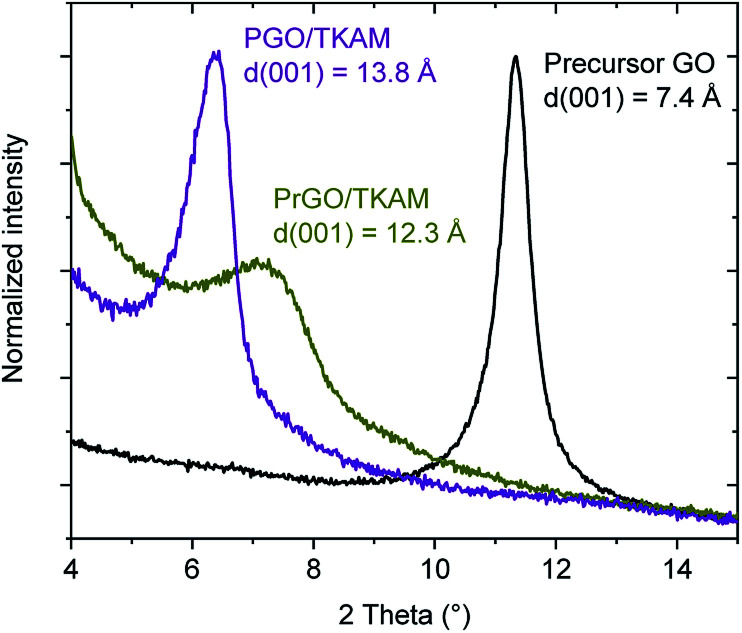
XRD patterns of precursor GO, PGO/TKAM and PrGO/TKAM in the angle region of (001) reflection.

Further evidence for the formation of pillared reduced GO material (PrGO/TKAM) was obtained using XRD, FTIR and analysis of nitrogen sorption isotherms. The position of (001) reflection in XRD patterns of GO allows to evaluate averaged distance between oxidized graphene sheets. The *d*(001) = 7.4 Å found for precursor GO at ambient humidity conditions increases up to 13.8 Å in PGO/TKAM thus providing expansion by ∼6.4 Å. This expansion exactly corresponds to the size of TKAM pillaring unit as demonstrated in an earlier study by structural modelling.^30^ XRD pattern recorded from PrGO/TKAM shows a slight decrease to *d*(001) = 12.3 Å (12.1 Å in second batch) but remains to be ∼5 Å higher compared precursor GO. Note that precursor GO heated above 200 °C decomposes into graphitic carbon^52^ which shows no peaks in the angle region shown in Fig. 3.

The thermal stability of PrGO/TKAM was confirmed also in a separate experiment with prolonged annealing at 280 °C for 12 hours. XRD test of the PrGO/TKAM sample still showed (001) peak of pillared rGO and absence of reflections from graphitic carbon (Fig. S2†). However, some background in the low angle region of the XRD pattern and increased broadness of the (001) reflection indicates some of loss of structural ordering.

The small decrease in *d*(001) observed as a result of heat treatment of PGO/TKAM can be explained either by loss of oxygen functionalities or partial loss of pillaring molecules. Annealing is also likely to assist in the evaporation of unreacted TKAM molecules trapped in GO lattice. Note that XRD analysis provides averaged value of interlayer distance in GO structure whereas gradual shifts in *d*(001) are typically interpreted in terms of random interstratification and intra-stratification.^12^ Therefore, removing some non-covalently bound pillaring TKAM molecules in process of annealing is plausible explanation for small downshift of the (001) peak position.

**Fig. 4:**
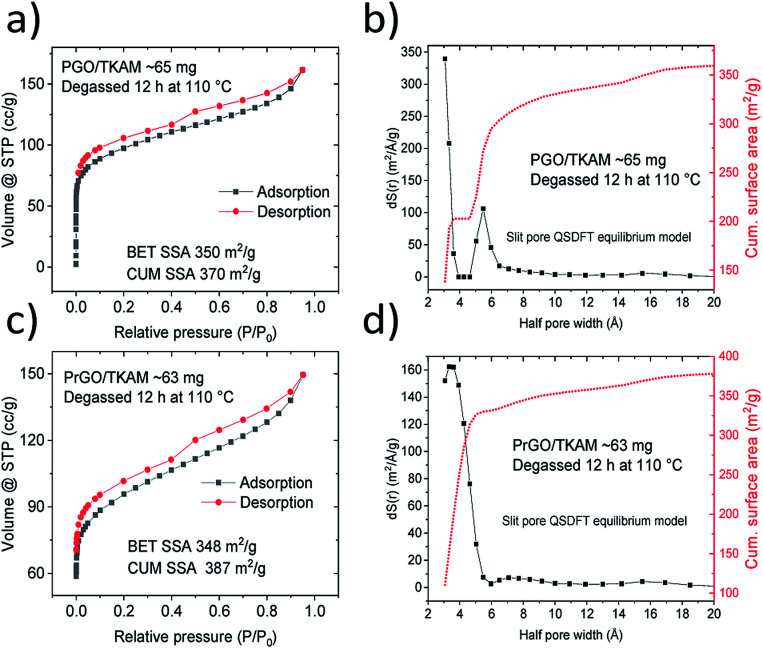
Nitrogen sorption isotherms recorded from PGO/TKAM before and after annealing at 280 °C (PrGO/TKAM).

Further evidence that the pillared structure remained intact after the annealing is provided by the analysis of nitrogen sorption isotherms (Fig. 4). Surface area of PGO/TKAM and PrGO/TKAM was estimated using both QSDFT slit pore model and the BET method. The data shown in Fig. 4 demonstrate that relatively high SSA of PGO/TKAM is preserved even after annealing at 280 °C. Similarly to precursor PGO/TKAM, the conditions of degassing have strong influence on the value of SSA determined by analysis of nitrogen sorption isotherms. Degassing of PrGO/TKAM at 110 °C (12 h) and 120 °C (24 h) resulted in BET SSA values of 350 m^2^ g^−1^ (DFT SSA 390 m^2^ g^−1^) and 400 m^2^ g^−1^ (DFT SSA 450 m^2^ g^−1^) respectively. The increase of SSA in this case is likely not connected to loss of oxygen as verified by XPS (C/O ∼ 4.1 for both degassing temperatures) and occurs solely due to better degassing of the PrGO/TKAM at higher temperature.

Remarkably, the pore size distribution of PrGO/TKAM is represented by a single peak originating from the pores with diameter ∼ 7 Å. That is in contrast with precursor PGO/TKAM that shows two peaks in the pore size distribution (Fig. 4).

**Fig. 5:**
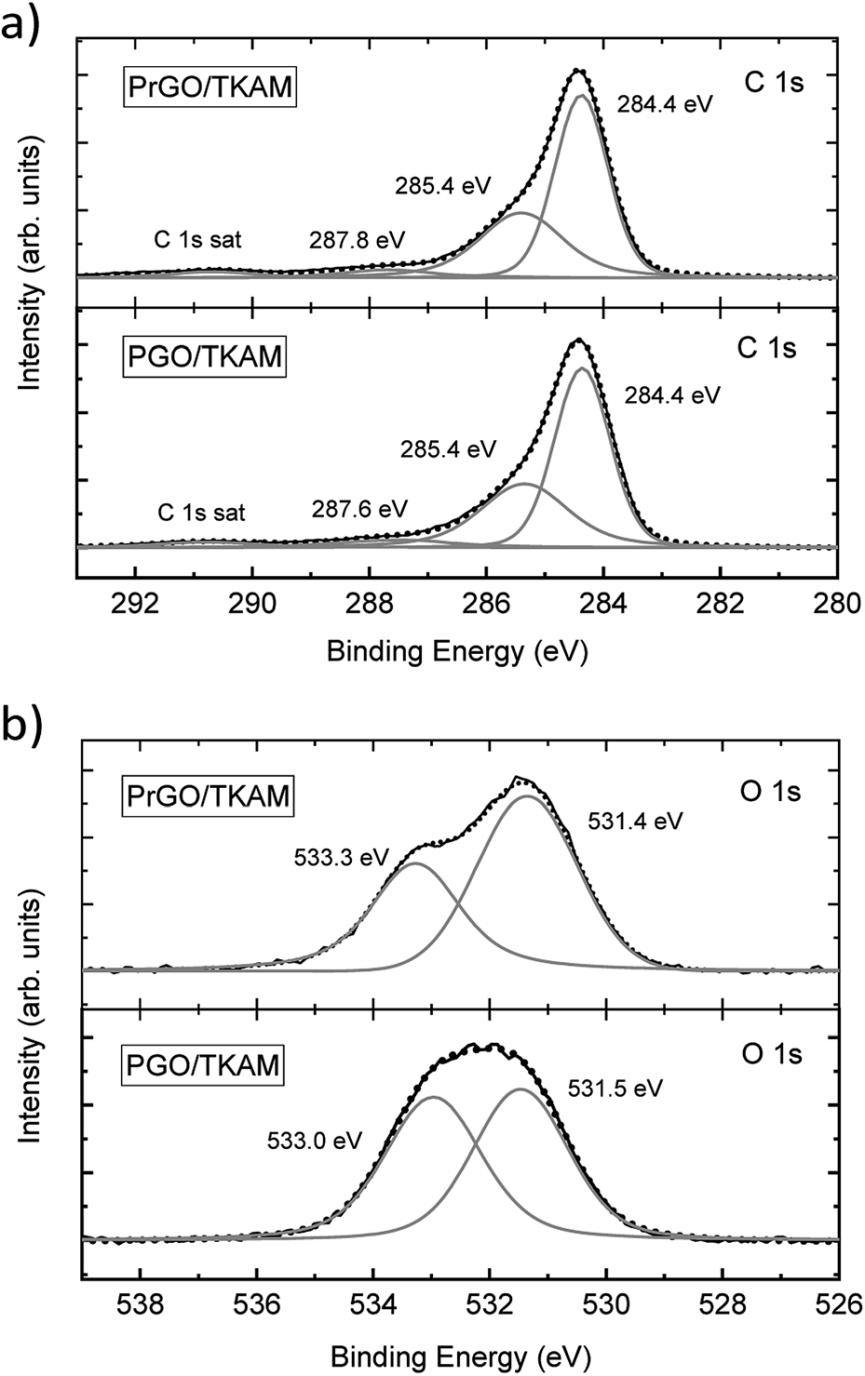
XPS of PGO/TKAM and PrGO/TKAM. (a) shows C1 s spectra, (b) shows O 1s spectra.

The pore size distribution observed for PrGO/TKAM is in agreement with the expected slit pore size of pillared rGO structure. Since the precursor PGO/TKAM is composed by oxidized graphene sheets pillared by TKAM molecules, annealing is expected to result in partial reduction and the formation of an extended network of unoxidized regions on individual graphene oxide sheets. Therefore, the dominant size of pores in the ideal structure of reduced PGO/TKAM can be estimated by subtracting van der Waals distance between pure graphene sheets in graphite (∼3.4 Å) from interlayer distance of PrGO/TKAM (12.3 Å). The value of ∼9 Å is in good agreement with pores size calculated using analysis of nitrogen sorption isotherms (7 Å) considering the well-known fact that thermal treatment does not remove all oxygen functional groups from graphene oxide. This general assumption is confirmed by chemical analysis of pillared GO materials performed using XPS (Fig. 5).

The XPS spectra of PGO/TKAM include peaks from carbon and oxygen, but also a clear signal from nitrogen (approximately 8.5 at%) thus confirming the presence of pillaring TKAM molecules. The relative number of pillaring molecules can be estimated using nitrogen concentration. The degree of GO oxidation can be estimated using the C/O ratio after excluding carbon atoms originating from TKAM molecules according to the formula C_25_N_4_H_24_ (N : C = 1 : 6.25). This yielded the C/O ratio 3.3 for PGO/TKAM, and 4.1 for PrGO/TKAM. Compared to precursor GO with C/O ratio 2.3, it is clear that both PGO/TKAM and PrGO/TKAM are partly reduced. However, PrGO/TKAM is reduced stronger than PGO/TKAM, as a result of thermal annealing. The C 1s spectra of PGO/TKAM and PrGO/TKAM are very similar (Fig. 5a) but the O 1s spectra (Fig. 5b) show certain difference in relative intensity of the main components.

Furthermore, reduction of GO in TKAM pillared structure is confirmed by improved conductivity of this material. Almost two order difference in conductivity of precursor PGO/TKAM and reduced material PrGO/TKAM was found (∼4 × 10^−5^ S m^−1^ and ∼1 × 10^−3^ S m^−1^) respectively.

**Fig. 6:**
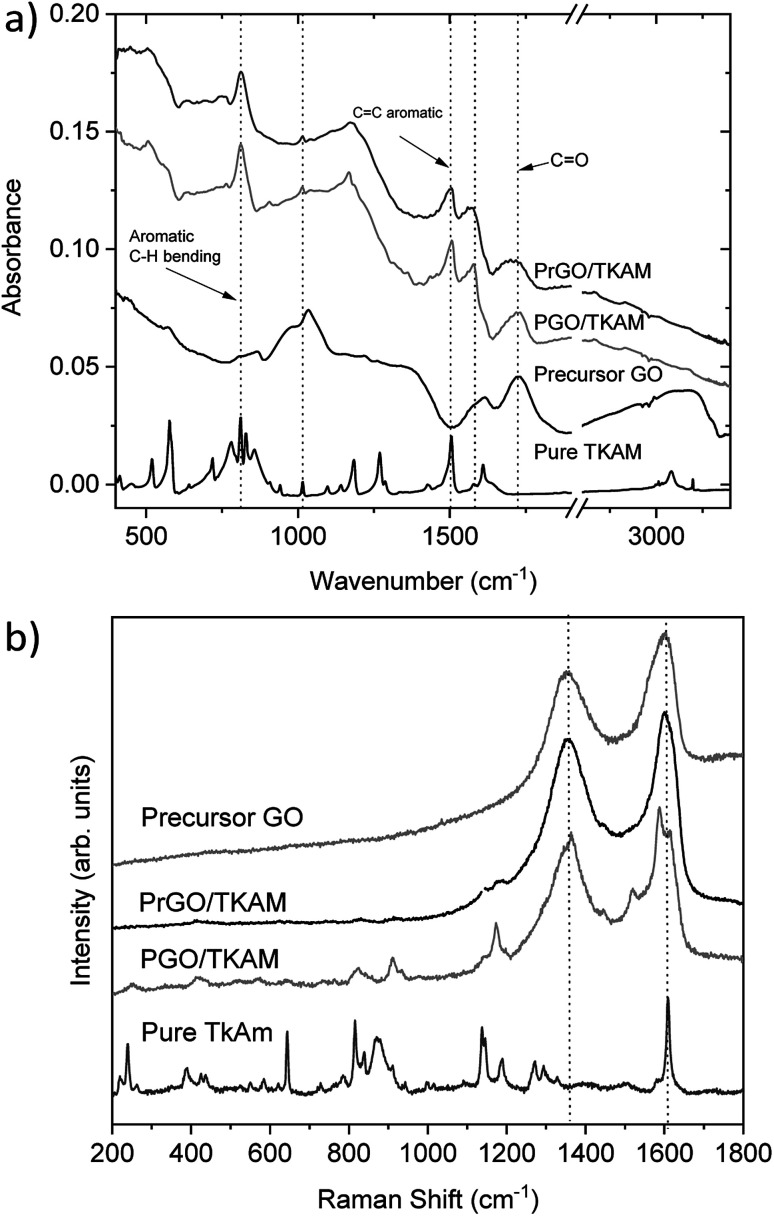
(a) FTIR spectra recorded from precursor GO, precursor TKAM powder, PGO/TKAM and PrGO/TKAM. The spectrum of pure TKAM has been scaled by factor 10. (b) Raman spectra of precursor TKAM powder, PGO/TKAM and PrGO/TKAM. The spectra for pure TKAM has been scaled down by factor 10 for easier comparison.

Further evidence of TKAM intercalation was obtained using FTIR and Raman spectroscopy (Fig. 6). The peaks related to reference TKAM can be observed in both PGO/TKAM and PrGO/TKAM. The spectra of PGO/TKAM show a combination of peaks from GO and TKAM as expected for pillared material. Some peaks related to TKAM can also be found in the spectra of PrGO/TKAM.

Reduction of PGO/TKAM is expected to result in a less hydrophillic material. The hydrophilicity of the materials was evaluated using quantitative analysis of water vapor sorption by Dynamic Vapor Sorption (DVS). Water sorption isotherms recorded using DVS provide change in the sample weight due to the sorption and desorption of water vapor. Using the BET equation for the water sorption isotherm it is possible to evaluate the specific surface area accessible by water (BET (H_2_O) SSA). The higher the BET (H_2_O) SSA the more hydrophilic is the material. Condensation of water inside of pores at higher vapor pressures can be detected in DVS isotherms by characteristic change of slope and quantified using onset value for the upturn in the sorption isotherm.

**Fig. 7:**
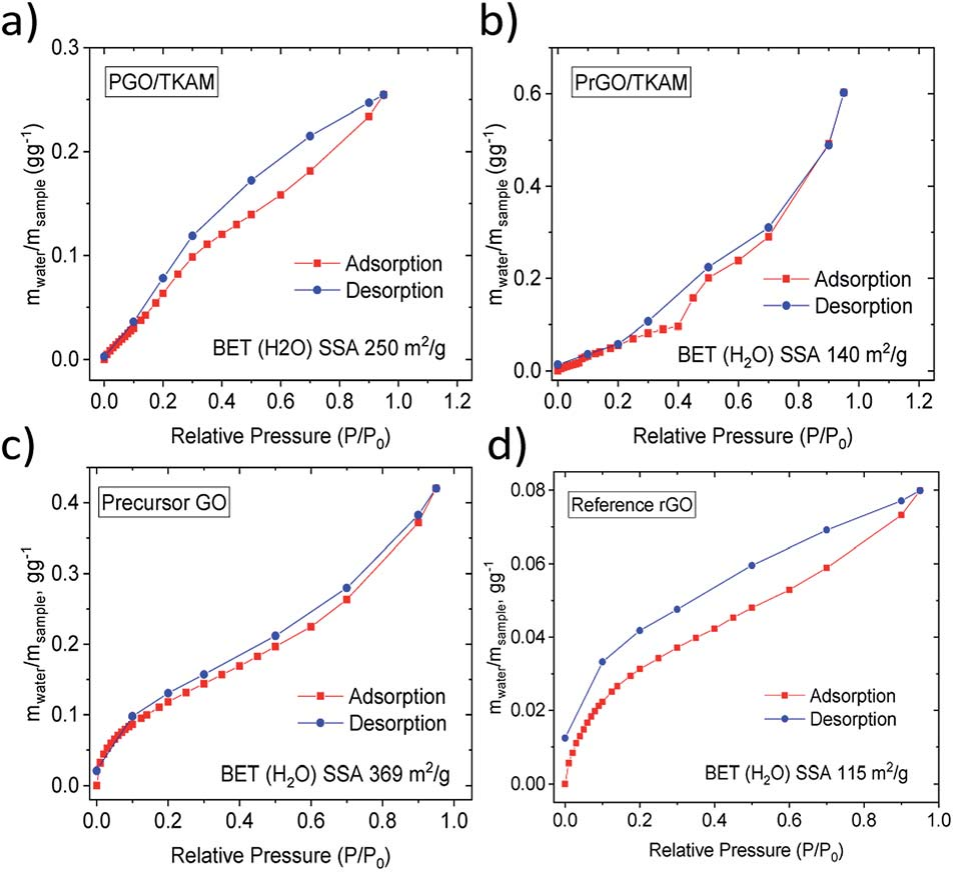
Water sorption and desorption isotherms recorded using DVS for precursor GO, reference rGO obtained by slow heating of GO, for PGO/TKAM GO and for PrGO/TKAM.

Water sorption isotherms recorded for precursor GO, reference rGO, PGO/TKAM and PrGO/TKAM are shown in Fig. 7. Precursor GO showed the highest BET (H_2_O) SSA of 345 m^2^ g^−1^ as expected for more hydrophilic material. Both PGO/TKAM and PrGO/TKAM showed smaller BET (H_2_O) SSA (250 m^2^ g^−1^ and 115 m^2^ g^−1^ respectively) as expected for partially reduced and less hydrophilic materials. Notably the reference sample of rGO obtained by thermal annealing of pure GO shows significantly smaller BET (H_2_O) SSA of 113 m^2^ g^−1^ compared to the PGO/TKAM, and distinctly different shape of sorption isotherm compared to the PrGO/TKAM. Graphitic rGO material does not exhibit any swelling. Therefore the sorption of water is limited only to the outer surface of flakes. In contrast, precursor GO absorbs most of the water between GO sheets thanks to strong swelling effect. For PGO/TKAM and PrGO/TKAM the mechanism of water vapor sorption is more complex as it is evident from the distinctly different isotherm shape with some change of slope rate around 35% *P*/*P*_0_ and different shape at lower values of *P*/*P*_0._ The shape of the isotherms indicates possible swelling effects but can also be related to simple sorption in slit pores of rigid pillared structure. Therefore we performed XRD experiments in order to verify the swelling properties of PGO/TKAM and PrGO/TKAM materials.

**Fig. 8:**
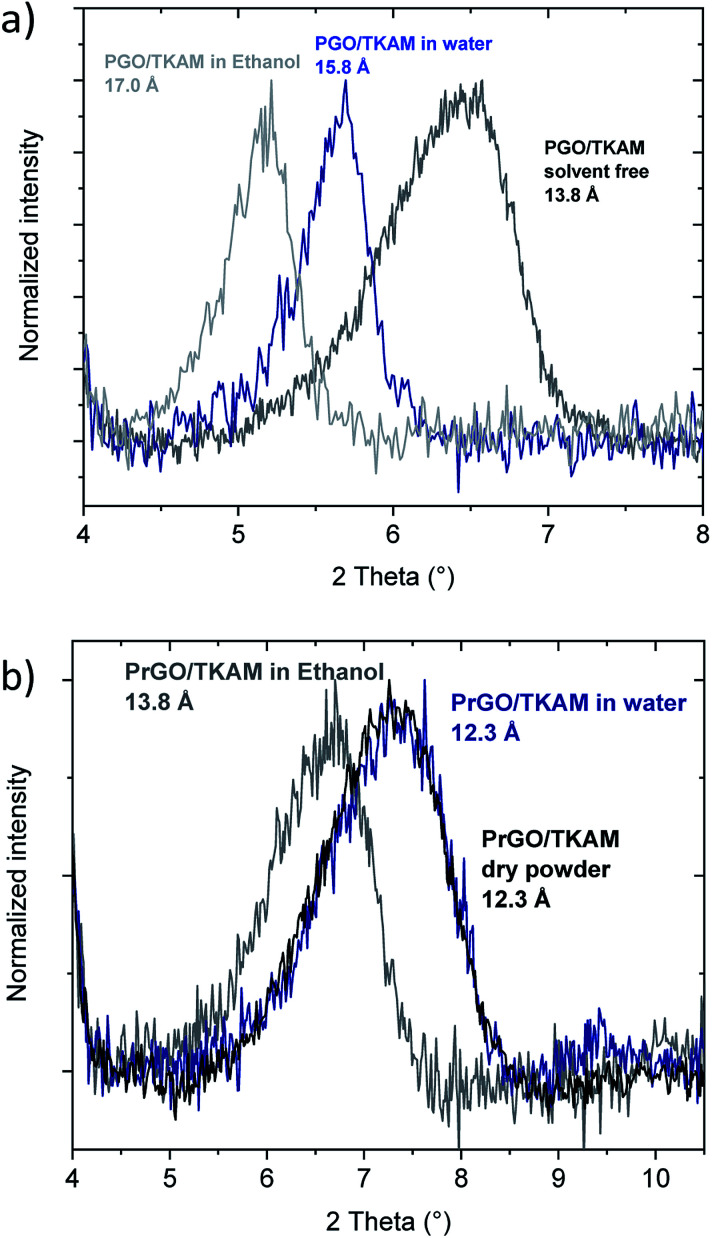
*In situ* XRD recorded from PGO/TKAM (a) and PrGO/TKAM (b) in excess of liquid water and ethanol.

The swelling of precursor PGO/TKAM in water and ethanol is somewhat weaker compared to graphite oxide^6,12,27,36^ but anyway quite pronounced (Fig. 8). The increase in *d*(001) spacing due to swelling in water (2 Å) corresponds approximately to the thickness of one water layer. Similar change in *d*(001)-spacing is observed for PGO/TKAM in excess of ethanol, where the change of ∼3.2 Å corresponds to thickness of one layer of ethanol. In contrast, no swelling is observed for PrGO/TKAM in water and rather minor increase of *d*(001) by ∼1.5 Å is found when the material is immersed in ethanol. It can be concluded that thermal reduction of PGO/TKAM results in almost complete loss of swelling properties in common polar solvents.

The difference in swelling properties of PGO/TKAM and PrGO/TKAM can be explained by suggesting that some of the TKAM pillaring molecules are not rigidly covalently bound to GO sheets in the precursor PGO/TKAM. Annealing at high temperatures not only help to remove some oxygen functionalities but also provides the possibility to interlink GO sheets by TKAM-based pillars.

The TKAM molecule has four amine groups and at least two of those could be connected to opposite sides of GO interlayer.

Three possible types of PGO/TKAM structures with high surface area were simulated in our previous study using theoretical modelling: (a) non-covalent intercalation of TKAM molecules (b), covalent attachment of TKAM molecules to GO sheets trough two of the four amine groups (c) and only one amino group reacted with GO thus attaching the molecule only to one side of GO interlayer. The modelling showed that all three structures cannot be reliably distinguished using XRD data due to rather similar interlayer distance of 14 Å to 15 Å for covalent and non-covalent pillaring. Therefore, swelling test is the most reliable source of information about possible presence or absence of interlinks between neighbouring GO sheets.

The nearly complete absence of swelling in PrGO/TKAM provides strong argument for covalently interlinked structure of this material and more hydrophobic nature of the PrGO/TKAM. It should be noted that swelling of multilayered GO is one of the major problems which hinders implementation of these materials for membrane applications. The ideal membrane material is expected to maintain the same size of permeation channels (provided by interlayer distance of GO) in all solvents and solutions. That is not the case for GO membranes which exhibit enormous difference in swelling when immersed in different solvents and solutions.^53,54^ Moreover the swelling is sensitive to ageing and modifies significantly after prolonged air-storage of membranes.^28^ Therefore, a lot of efforts were recently aimed on the preparation of GO membranes with negligible swelling.^17,55–57^ Using PrGO/TKAM for the preparation of membranes with reduced swelling can be proposed as a plausible alternative.

Summarizing the data presented above, thermal annealing of TKAM pillared GO results in the reduction of GO sheets but preserves relatively high surface area by nitrogen sorption and expanded GO lattice.

Therefore, we demonstrate that the annealing of PGO/TKAM results in the formation of TKAM pillared rGO in full accordance to the definition of pillared structure provided in the introduction section. The evidence provided for PrGO/TKAM by XRD, XPS, N_2_ and H_2_O sorption isotherms, FTIR and Raman spectra confirms the presence of TKAM-based pillaring units, expanded GO lattice, relatively high surface area, decreased swelling and sorption of water as expected for more hydrophobic material thanks to thermal reduction.

## 4. Conclusions

In conclusion, we demonstrate that TKAM pillared GO can be converted into TKAM pillared reduced GO using mild thermal annealing. Thanks to remarkable thermal stability of TKAM pillaring units the porous structure formed by pillared graphene oxide sheets preserves after thermal treatment. The GO lattice remains expanded thanks to TKAM pillars while the interconnected pore network provides a path for escape of the gases formed in the process of thermal deoxygenation of graphene oxide. The PrGO/TKAM can be considered as one of few examples of true pillared rGO materials and the first one obtained using pillared GO as a precursor. Rather uniform and narrow pore size distribution of PrGO/TKAM with single peak at ∼7 Å slit pore diameter makes it promising for membrane applications. However, stronger reduction is needed to further improve conductivity of this material *e.g.* for application as porous electrode in supercapacitors.

## Conflicts of interest

There are no conflicts of interest to declare.

## Supplementary Material

RA-010-D0RA00067A-s001
