# Supplementary material for: Thermally reduced pillared GO with precisely defined slit pore size
Source: RSC Adv. 2020 Feb 13;10(12):6831–9. doi: 10.1039/d0ra00067a (PMC9049709; doi:10.1039/d0ra00067a)
Supplement: RA-010-D0RA00067A-s001 [file RA-010-D0RA00067A-s001.pdf]

Electronic Supplementary Material (ESI) for RSC Advances.
This journal is © The Royal Society of Chemistry 2020

Supplementary Information

Thermally reduced pillared GO with precisely defined slit pore size

Andreas Nordenström,[a] Artem Iakunkov, [a] Jinhua Sun [a,b] and Alexandr V. Talyzin *[a]

1. Additional characterization of materials.

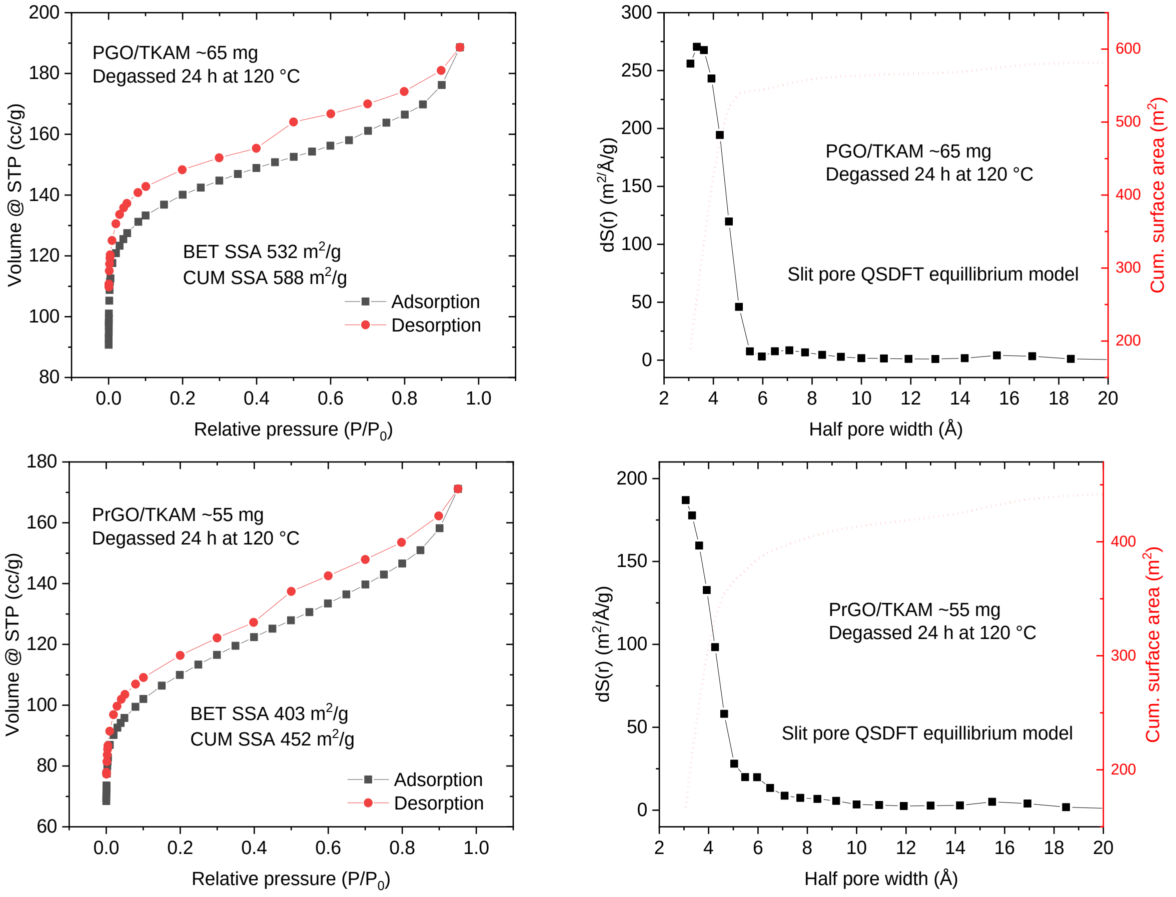

Figure S1 Isotherms of PGO/TKAM and PrGO/TKAM for samples outgassed at 120 °C for 24 h

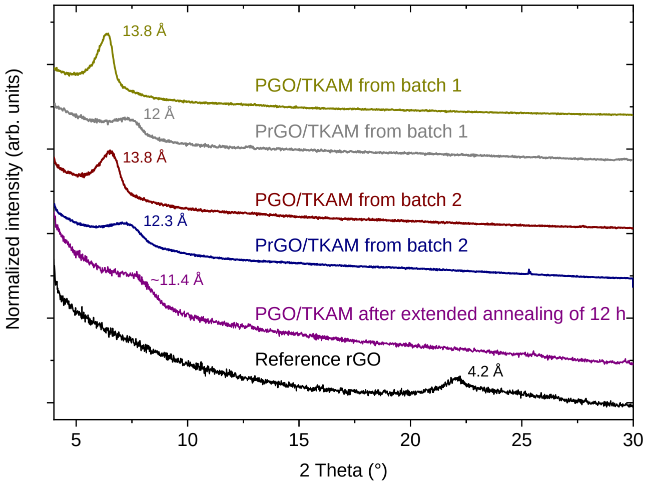

Figure S2 XRD patterns recorded from two batches of PGO/TKAM and PrGO/TKAM, reference rGO and PGO/TKAM after12 hours of annealing

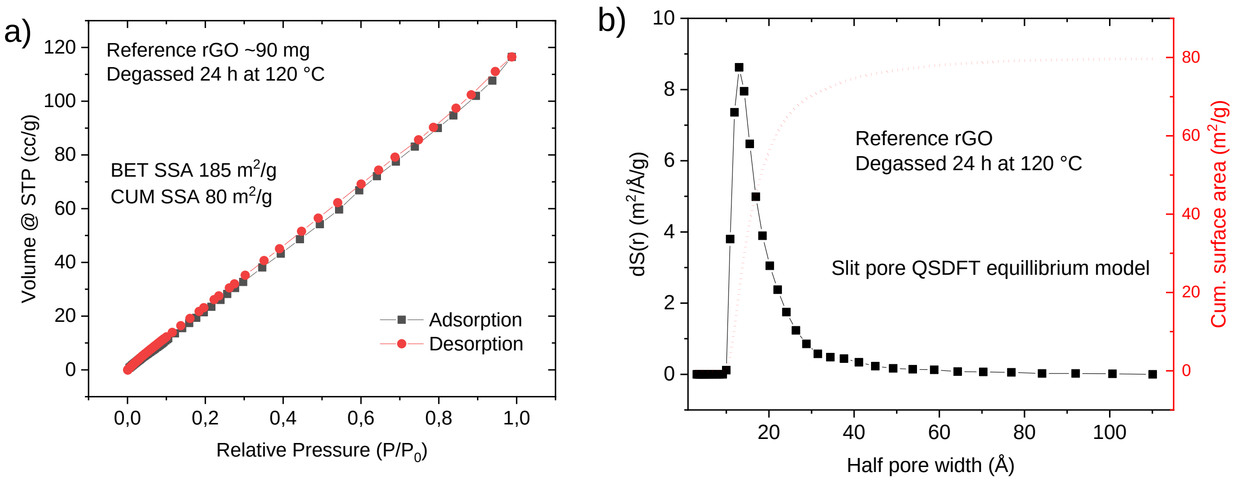

Figure S3 Nitrogen sorption isotherm (a) and pore size distribution (b) for reference rGO

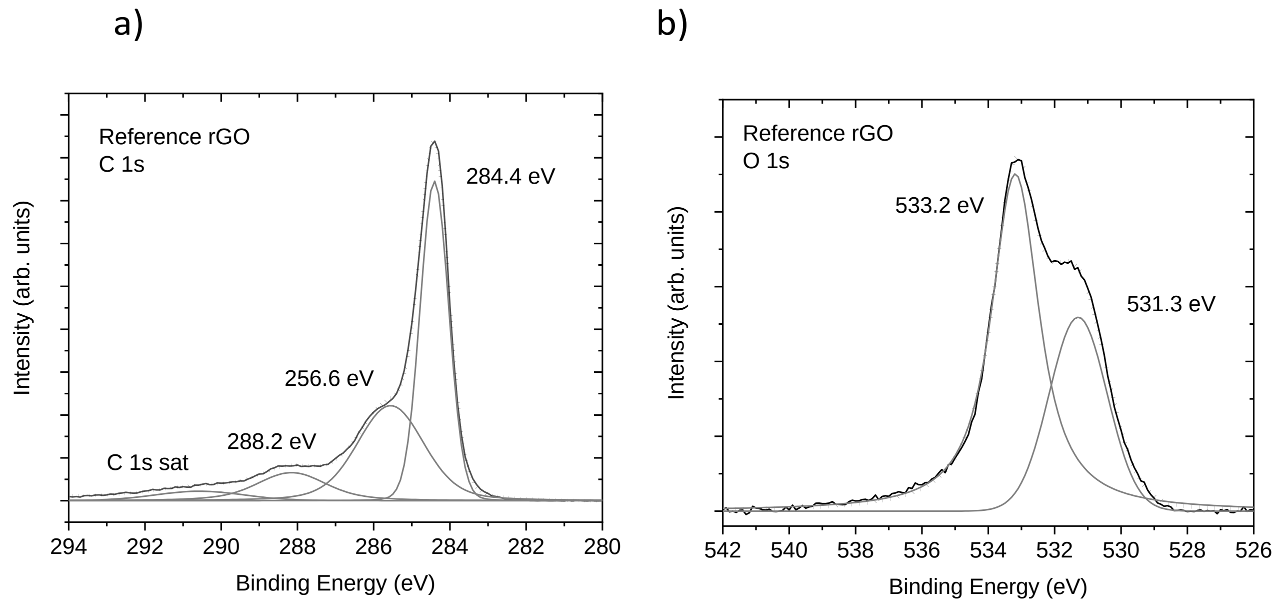

Figure S4 XPS for reference rGO (C/O = 5.1): a) C1s and b) O1s

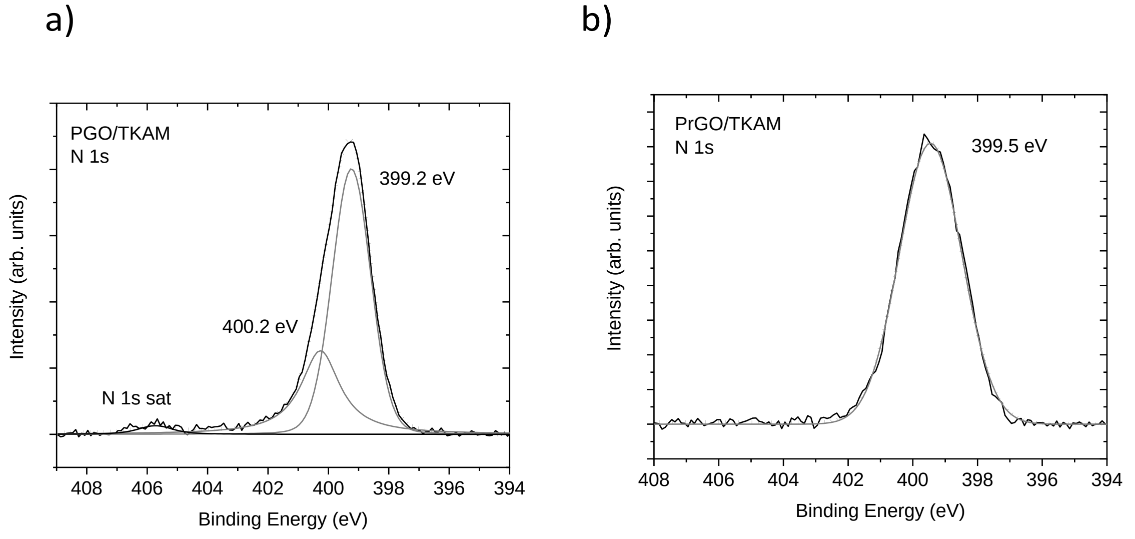

Figure S5 XPS N 1s spectra for PGO/TKAM (a) and PrGO/TKAM (b)

a)

b)

c)

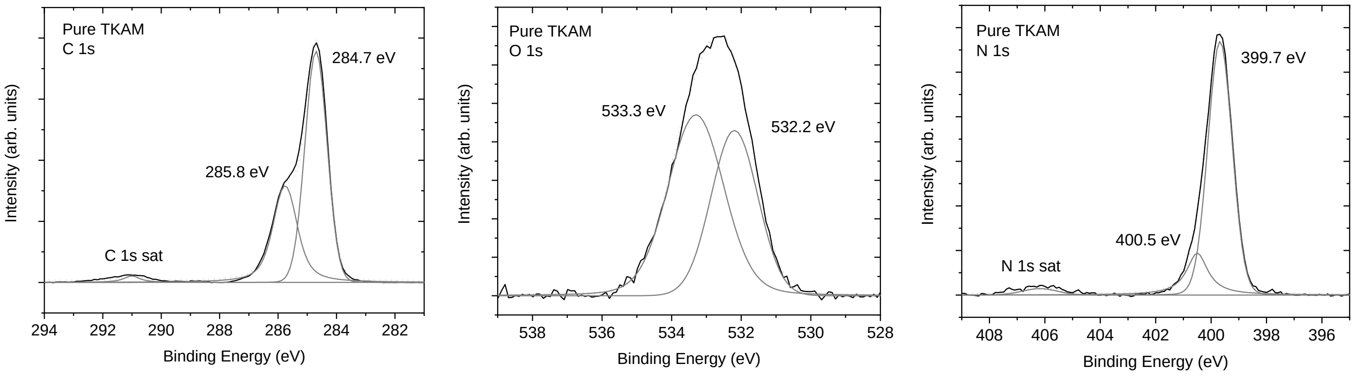

Figure S6 XPS spectra of pure TKAM:): a) C1s and b) O1s
